# Spatiotemporal dynamics of different CO_2_ fixation strategies used by prokaryotes in a dimictic lake

**DOI:** 10.1038/s41598-019-51584-0

**Published:** 2019-10-21

**Authors:** Albin Alfreider, Barbara Tartarotti

**Affiliations:** Department of Ecology, University of Innsbruck, Innsbruck, Austria

**Keywords:** Limnology, Microbiology

## Abstract

The Calvin-Benson-Bassham (CBB) cycle and the 3-hydroxypropionate/4-hydroxybutyrate (HP/HB) cycle are two inorganic carbon assimilation pathways widely used by prokaryotic autotrophs in lakes. We investigated the effect of mixing periods and stable water stratification patterns on the trajectories of both CO_2_ fixation strategies in a dimictic lake (Piburger See), because information on the spatiotemporal dynamics of prokaryotes using these pathways in freshwater ecosystems is far from complete. Based on a quantitative approach (droplet digital PCR) of genes coding for key enzymes in different CO_2_ assimilation pathways, nine depths covering the entire water column were investigated on a monthly basis for one year. Our data show that the abundance of photoautotrophs and obligate chemolithoautotrophs preferentially using form IA RubisCO was determined by seasonal variations. Highest numbers were observed in summer, while a strong decline of prokrayotes using RubisCO form IA was measured between December and May, the period where the lake was mostly covered by ice. The spatiotemporal distribution patterns of genes coding for RubisCO form IC genes, an enzyme usually used by facultative autotrophs for CO_2_ assimilation, were less pronounced. Bacteria harboring RubisCO form II were dominating the oxygen limited hypolimnion, while nitrifying Thaumarchaeota using the HP/HB cycle were of minor importance in the lake. Our data reveal that the seasonal heterogeneity, which is determined by the dimictic thermal regime of the lake, results in pronounced spatiotemporal changes of different CO_2_ assimilation pathways with depth-dependent environmental parameters as key factors for their distribution.

## Introduction

Primary production based on photosynthesis performed by plants, algae, cyanobacteria and anoxygenic bacteria is a major biological process on earth and of fundamental importance in the global carbon cycling^1^. The presence of light, however, is not a prerequisite for the assimilation of inorganic carbon, as also chemosynthesis can provide energy for this process. The chemoautotrophic fixation of inorganic carbon is accomplished by diverse prokaryotes and biochemical variations among different pathways suggest that this capability evolved convergently^2–4^. To date, six mechanisms are known by which autotrophic organisms fix carbon^3^, whereby the Calvin-Benson-Bassham (CBB) cycle in bacteria and the recently discovered 3-hydroxypropionate/4-hydroxybutyrate (HP/HB) cycle in Archaea are widely distributed in the pelagic environment of marine^4,5^ and freshwater habitats^6,7^.

A CO_2_ fixation mechanism used by a variety of organisms is the CBB pathway^8,9^. The enzyme responsible for the actual fixation of CO_2_ in the Calvin cycle, ribulose-1,5-bisphosphate carboxylase/oxygenase (RubisCO), is not only used by photoautotrophs, different forms of RubisCO also occur in ecologically and evolutionary diverse chemoautotrophic bacteria^8^. Phylogenetic analyses based on large subunit genes and deduced amino acid sequences divide form I RubisCO into two groups (“green” and “red”), which may be further subdivided into types IA, IB, IC, ID^8,9^. Bacterial genome sequences indicate that there are even two different types of form IA structures, with form IAc being associated with carboxysomes^8^. Cynaobacteria and obligate chemolithoautotrophs often possess form IA RubisCO, whereas form IC enzymes are often associated with facultative autotrophs^8,10^. The form II RubisCO enzyme is markedly different from form I. Important biochemical features of form II enzymes are their poor affinity to CO_2_ and the low specificity factor τ, a measure of the ability of the RubisCO to discriminate between CO_2_ and O_2_^11^. Therefore, RubisCO form II enzymes are adapted to environments characterized by low oxygen concentrations. A variant of the HP/HB cycle operates in nitrifying Thaumarchaeota^12^. This pathway was described as the most energy efficient aerobic carbon fixation cycle. This characteristic of the pathway and physiological adaptations to very low ammonia concentration correspond to the oligotrophic lifestyle of Thaumarchaeota^13^.

Stratified lakes represent ideal systems to investigate proximate physical and chemical drivers of autotrophic microbial communities in a relatively stable framework of habitat heterogeneity^6^. Chemolithoautotrophy is often depending on oxygen-labile compounds as electron donors and therefore this metabolism is often detected along chemical gradients and in the oxic/anoxic interfaces, which can be particularly found in meromictic and/or eutrophic lakes. Several studies have already shown that bacteria using different forms of RubisCO in the CBB pathway are widely distributed in different lake types and climate zones^6,14–18^. Thaumarchaeota using the HP/HP cycle are the dominant group of chemoautotrophs in oligotrophic and deep lakes. However, most studies investigating nitrifying Thaumarchaeota in lakes were targeting the ammonia oxidation potential, based on the analysis of genes coding for ammonia monooxygenase^19–23^. Investigations focusing on autotrophy related genes are limited to a few studies^6,7,24,25^.

Although autotrophic prokaryotes play a key role in biogeochemical cycling of nitrogen, sulphur and other elements in lakes, information on seasonal succession of autotrophs using different CO_2_ fixation strategies is rare. Accordingly, the goal of this investigation was to examine the spatiotemporal trajectories of autotrophic prokaryotes using different CO_2_ assimilation pathways in Piburger See, focusing on chemoautotrophic organisms. A previous investigation in seasonally stratified lake, based on a single sampling event during summer stratification, revealed a high diversity of sequences coding for three forms of RubisCO in the CBB cycle^6^. For the current study, we were especially interested to follow the spatiotemporal variations of genes coding for different forms of RubisCO and to examine if the occurrence of these forms is related to seasonal changes of environmental variables. Another specific objective was to evaluate if the lack of autotrophic Thaumarchaeota during summer^6^ also occurs under ice during winter, when environmental conditions for chemoautotrophic growth are more favourable. For the current investigation, water samples were obtained from nine depths sampled on a monthly basis over one year. Gene abundances were analysed via a set of autotrophic gene markers coding for key enzymes in the different CO_2_ assimilation pathways based on droplet digital PCR (ddPCR). Functional genes were correlated with limnological observations and environmental parameters in order to identify which physical and chemical drivers and processes are most influential for their quantitative occurrence.

## Results and Discussion

### Seasonal environmental changes

**Figure 1:**
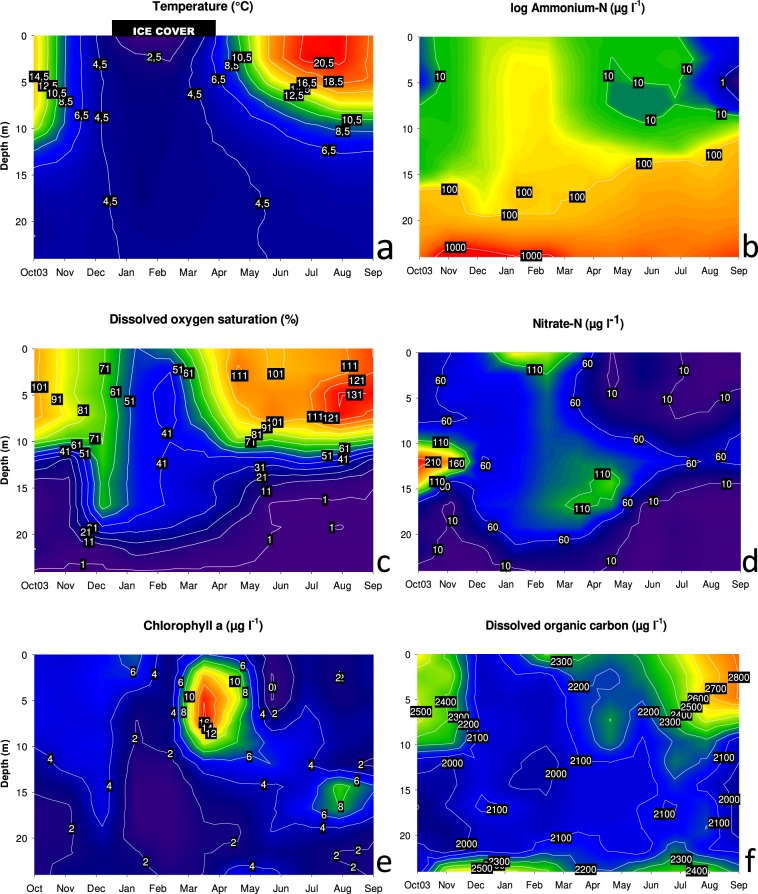
Spatiotemporal dynamics of temperature (**a**), ammonium (**b**), dissolved oxygen saturation (**c**), nitrate (**d**), chlorophyll *a* (**e**), and dissolved organic carbon (**f**) measured monthly over a period of one year. Please note the logarithmic scale in the ammonium plot. The black shaded rectangle on the top of the temperature plot represents the approximate duration of the ice cover.

Stratification of Piburger See followed a typical seasonal pattern for a dimictic lake: autumnal mixis occurred at the end of November 2016 and spring thermal overturn shortly after the disappearance of the ice cover at the end of March 2017 (Fig. 1a). An inverse stratification was observed during winter under the ice cover, while from April to early November a stable summer stratification developed. The surface water temperature reached a maximum of 21.1 °C in August. Dissolved oxygen (DO) concentrations suggest that both thermal overturns are very short and not complete, producing a non-mixing water body (monimolimnion) below 21 m depth (Fig. 1c). In the hypolimnion, oxygen was strongly depleted during summer stratification with <10% DO saturation between June and November in depths up to 15 m. Anoxic conditions were observed at the deepest sampling point (24 m) from October to May. From June to September, the anoxic zone reached up to 18 m depth. In the surface water, DO supersaturation was observed during summer stratification with a maximum of 142% in August at 6 m depth. Nitrate concentrations showed the highest values (230 µg NO_3_-N l^−1^) in October in the upper hypolimnion (Fig. 1d). In February, under ice, nitrate concentration reached up to 166 µg NO_3_-N l^−1^ close to the lake surface. This peak was presumably caused by in-lake nitrification as described for northern oligotrophic and mesotrophic lakes with a winter ice cover^26^. Nitrate depletion, presumably caused by photoautotrophs, occurred in the epilimnion of the lake. In the hypolimnion, nitrate generally decreased with depth, associated with a pronounced accumulation of ammonium in the reducing environment at the deepest sampling depths (Fig. 1b). Dissolved organic carbon (DOC) measurements demonstrated low spatiotemporal variation, with concentrations constantly higher than 2 mg 1^−1^. Maximum values were reached in September in the surface water sample (2.82 mg DOC l^−1^, Fig. 1f). Chlorophyll *a* (Chl *a*) levels peaked at the sampling date in April in the epilimnion (Fig. 1e), most probably caused by an algal spring bloom that occurs regularly in Piburger See at this time of the year^27–29^. The increase of Chl *a* in the hypolimnion in late summer was also observed in other years and interpreted as a result of sedimentation and light adaptation processes of the photoautotrophic plankton during summer stratification^27^.

### Vertical and temporal distribution of different forms of RubisCO in the Calvin cycle

Autotrophic gene abundance coding for three forms of RubisCO (IA, IC, II) was determined to follow the spatiotemporal dynamics of bacteria using the Calvin cycle for the assimilation of inorganic carbon (Table 1).

**Table 1:** Specification of primers applied for the detection of different functional genes related to autotrophic prokaryotes based on ddPCR.

Pathway, Targeted protein, Phylogenetic coverage	Primer Name	Sequence (5′-3′)	Pconc^a^	Ta^b^	Reference
**CBB-cycle**					
RubisCO form IA	cbbL_IA_f	CGGCACSTGGACCACSGTSTGGAC	200	59	Alfreider *et al*.^43^
*Proteobacteria (α, β, γ), Actinobacteria, Cyanobacteria*	cbbL_IA_r	GTARTCGTGCATGATGATSGG			
RubisCO form IA in chemoautotrophs	cbbL_IA_CHEM	GARGGNTCNGTNGTYAACGT	400	58	Alfreider & Bogensperger^30^
*Proteobacteria (α*, *β*, *γ)*, *Actinobacteria*	cbbL_IA_r	GTARTCGTGCATGATGATSGG			
RubisCO form IC	cbbLR1F	GAACATCAAYTCKCAGCCCTT	200	57	Alfreider *et al*.^34^
*Proteobacteria (α*, *β*, *γ)*, *Actinobacteria*	cbbLR1R	TGGTGCATCTGVCCGGCRTG			
RubisCO form IC dominant in lakes	cbbL_IC_lake_f	AGCCGTMARYAAAGCCAGC	200	60	This study
*Burkholderiales*	cbbL_IC_lake_r	CGTARAARCCSCGGATCATC			
RubisCO form II	cbbM_f	GGCACCATCATCAAGCCCAAG	200	53	Alfreider *et al*.^43^
*Proteobacteria (α, β, γ), Dinophyceae*	cbbM_r	TCTTGCCGTAGCCCATGGTGC			
**3-HP/4-HB cycle**					
4-hydroxybutyryl- CoA dehydratase	qPCR_hcd_f	GACTGATCCWAAAGGDGAYAGAAG	250	56	Alfreider *et al*.^6^
*Thaumarchaea* 1.1a	qPCR_hcd_r	CCYTTARCATCTGCWGGAATTGC			
**Ammonia oxidation pathway**					
Ammonia monooxygenase	GenAOAF	ATAGAGCCTCAAGTAGGAAAGTTCTA	100	55	Meinhardt *et al*.^40^
*Thaumarchaea*	GenAOAR	CCAAGCGGCCATCCAGCTGTATGTCC			

^a^Primer concentration (µmol); ^b^Ta: Annealing temperature (°C).

#### RubisCO form IA

**Figure 2:**
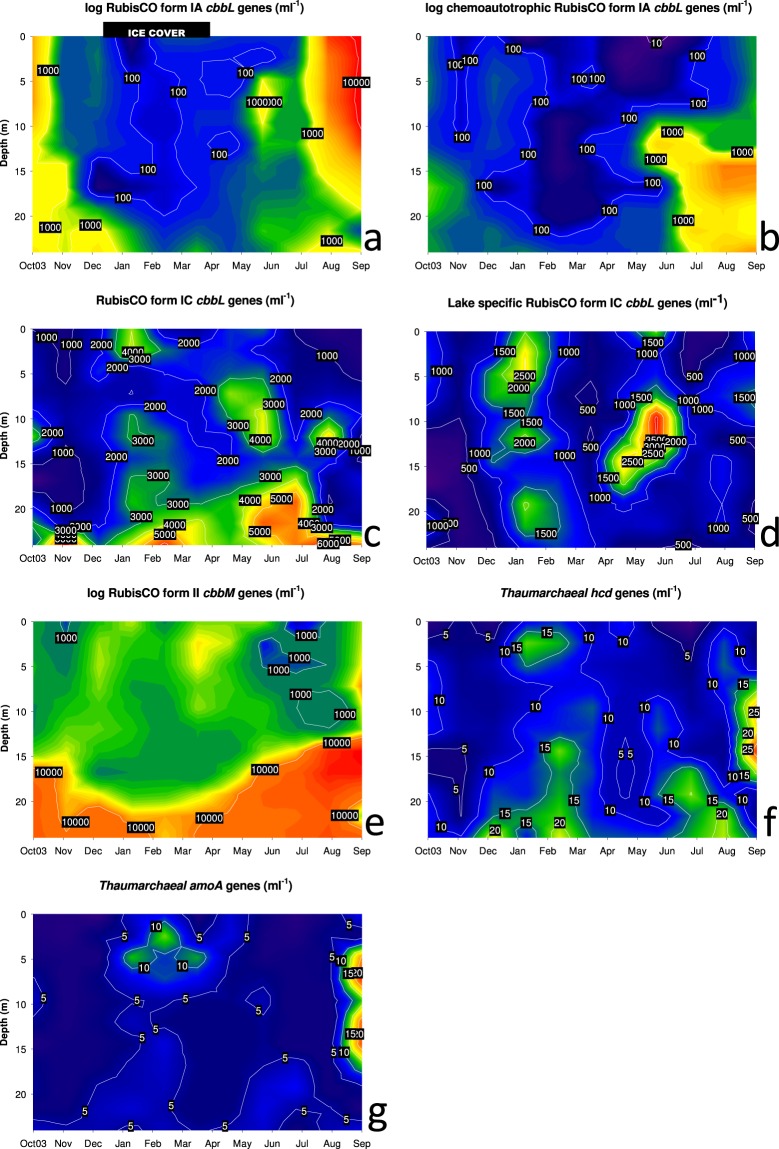
Spatiotemporal dynamics of *cbbL* (targeting different RubisCO IA and IC forms; **a**–**d**), *cbbM* (targeting RubisCO form II; **e**) and thaumarchaeal (*hcd*, *amoA*; **f**, **g**) gene copy numbers. Samples for analysis were taken monthly along a vertical profile at 3-m intervals. Please note the logarithmic scale in the *cbbL*-form IA and *cbbM* plots.

RubisCO form IA (cbbL-IA) gene numbers showed a pronounced maximum in the epilimnion during late summer stratification (Fig. 2a), which was most likely affiliated with cyanobacterial lineages harboring form IA of RubisCO. Sequence analysis of *cbbL*-IA genes performed in former studies^6,30^ in Piburger See and other lakes suggest that these RubisCO genes are affiliated with *Synechococcus* spp. and *Paulinella chromatophora* chloroplasts. The dominance of coccal cyanobacteria at this time of the year was also observed in an additional investigation, which was focusing on the seasonal succession of the phytoplankton community in Piburger See^31^. In this study, taxonomic assignments were based on morphological identification and *Snowella litoralis*, *Aphanothece clathrata*, as well as *Microcystis incerta* were the most abundant species. Gene abundances based on ddPCR analysis with primer cbbL_IA_CHEM, which was designed to encompass a functionally wide range of bacterial chemolithoautotrophs and also provides selectivity against oxygenic phototrophs using RubisCO form IA for CO_2_ fixation (Table 1)^30^, also peaked during late summer stratification. However, in contrast to the results obtained by the broad range primer pair for *cbbL* form IA genes, chemoautotrophic RubisCO form IA showed highest values in the hypolimnion of the lake (Fig. 2b). Spatiotemporal distribution patterns of *cbbL*-IA gene abundances based on both primer pairs showed a similar trend in winter: numbers declined during winter stagnation within almost the entire water column and stayed at a very low level until spring. In former studies, phylogenetic analysis in a variety of lakes including Piburger See showed that chemoautotrophic representatives were mostly Betaproteobacteria^6,30^, including a considerable part of *cbbL*-form IA sequences affiliated with the *Nitrosomonas oligotropha* lineage (cluster 6a). A second major cluster was related to the hydrogen-utilizing *Comamonadaceae* bacterium H1. Sequence analysis performed with cbbL_IA_CHEM primer additionally revealed a phylotype closely related to *Thiobacillus denitrificans*, which was detected in 9 m depth during summer stagnation^30^.

#### RubisCO form IC

**Figure 3:**
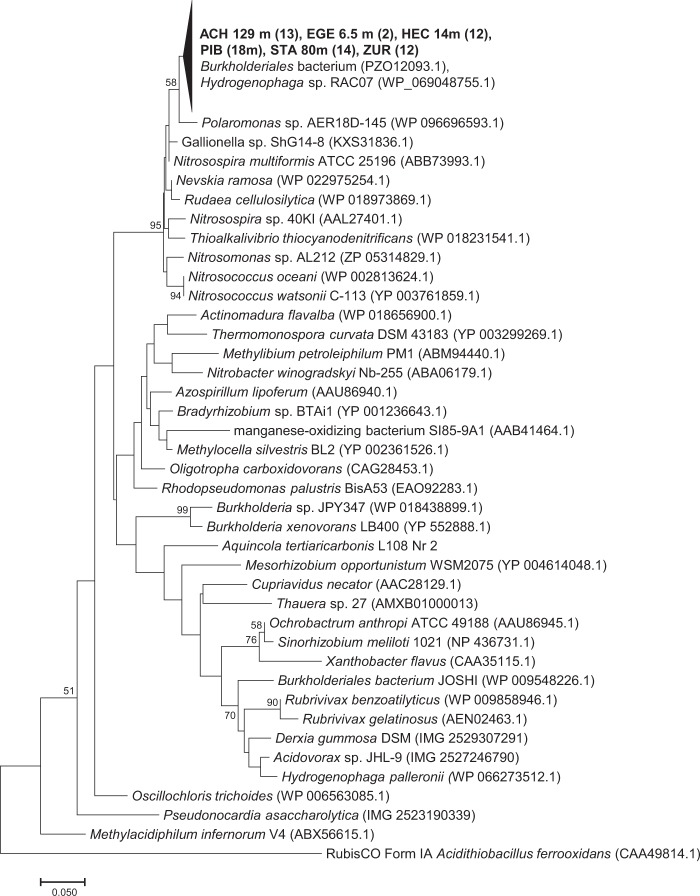
Phylogenetic tree reflecting the coverage and specificity of qPCR primers cbbl_IC-lake targeting RubisCO form IC in *Burkholderiales* based on amino acid sequence analysis (shown in bold) and representative relatives obtained from GeneBank. DNA was extracted from water samples of different lakes obtained in a former study (Achensee - ACH, Egelsee - EGE, Hechtsee - HEC, Piburger See - PIB, Starnberger See - STA; Zürichsee - ZUR; Alfreider *et al*.^6^. Numbers in brackets indicate the number of clones analyzed.

In surface water, bacteria harbouring RubisCO form IC showed maximum values in February under ice and in June and August below the metalimnion. The highest *cbbL* numbers, however, were detected in the lower hypolimnion throughout the year (Fig. 2c). In agreement with form IA sequences, Alfreider *et al*.^6^ showed that based on phylogenetic analysis of form IC sequences Betaproteobacteria were also the taxonomic dominate group of autotrophs. One major clade with highly similar sequences related to different *Burkholderiales* representatives was detected in a wide range of different lakes and depths including Piburger See^6^. In order to quantify this important group of autotrophs, for the present work primer pair “cbbL_IC-lake” specifically targeting this cluster of form IC RubisCOs was successfully designed and tested (Fig. 3). Determination of gene abundances related to RubisCO form IC “lake” were in accordance with measurements retrieved with broad range primers of Form IC in February under ice and in May/June at depths between 9 and 15 m (Fig. 2d), indicating that at these depths and dates of the year representatives of the lake cluster were the dominating group of bacteria using RubisCO form IC for CO_2_ fixation. Information on the ecological properties of the lake cluster bacteria can be derived from the closest cultivated relatives within this cluster (Fig. 3): *Hydrogenophaga* sp. RAC07, which was isolated from the phycosphere of *Chrysochromulina tobin,* and a *Burkholderiales* microbiome obtained from non-axenic cyanobacterial cultures and an associated *Burkholderiales* microbiome^32,33^ suggest that members of the RubisCO_form_IC-lake cluster are associated with photoautotrophic planktonic organisms. Furthermore, *Hydrogenophaga* sp. RAC07 was originally cultivated on organic carbon substrates supporting heterotrophic growth^33^, proposing that this bacterium is a facultative autotroph exhibiting a metabolic flexibility in relation to the carbon source. In the hypolimnion, cbbL_IC_lake numbers did not correspond with maxima observed by genes detected with broad range primer for Form IC RubisCO. Based on the wide diversity of organism harbouring RubisCO form IC (Fig. 3), the function and taxonomic identity of bacteria not associated with the form IC lake cluster in the hypolimnion remain unclear.

**Figure 4:**
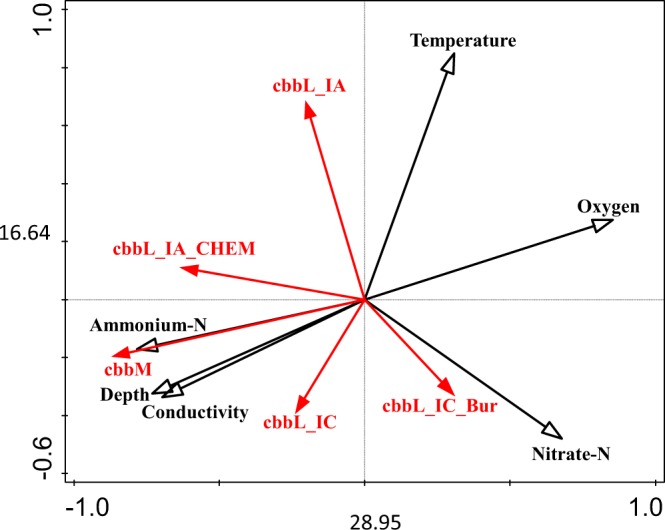
Redundancy analysis biplot of the influence of selected environmental parameters (black arrows) on abundances of different RubisCO genes (*cbbL*_IA = RubisCO form IA; *cbbL*_IA_CHEM = RubisCO form IA in chemoautotrophs, *cbbL*_IC = RubisCO form IC; *cbbL*_IC_lake = RubisCO form IC in lakes; *cbbM = *RubisCO form II) obtained from water samples of Piburger See (shown in red, n = 108). Copy numbers of thaumarchaeal genes (*hcd*, *amoA*) were not analysed due to the low variability of the data close to the detection limit.

In contrast to RubisCO form IA gene levels, which showed distinct seasonal and depth-dependent distribution patterns, abundance range and seasonality of organism harbouring form IC genes were less pronounced (Fig. 2a–d). These differences can be explained by the functional diversity of autotrophs using these forms of RubisCO. Most obligate chemolithoautotrophs possessing form IA RubisCO are highly specialized in their physiology and therefore adapted to narrow temporal and spatial ecological niches in the lake. Form IC enzymes, on the other hand, are often associated with facultative autotrophs with an enhanced metabolic flexibility in the utilization of different carbon sources^8^, resulting in a broader spatial and temporal distribution. This could also explain that the occurrence of bacteria using the RubisCO form IC for CO_2_ fixation in Piburger See were not clearly associated with individual environmental parameters as revealed by RDA-analysis (Fig. 4).

#### RubisCO form II

Throughout the year, highest abundances of RubisCO form II were observed under anoxic and microaerobic conditions in the hypolimnion of the lake (Fig. 2e). Correspondingly, the distribution of *cbbm* numbers was correlated with depth-dependent environmental parameters with a negative correlation observed with DO (Fig. 4). A pronounced maximum was observed in August and September below 15 m depth. The strong influence of DO on the distribution of *cbbM* can be explained by a characteristic biochemical feature of the RubisCO form II protein: a poor affinity to CO_2_ and a low CO_2_/O_2_ substrate specificity^9^. On average, *cbbM* was the most abundant autotrophic gene marker observed in Piburger See (avg 6.78 × 10^3^ genes ml^−1^, n = 106), about three times more numerous than *cbbL*-IC (avg 2.39 × 10^3^ genes, n = 106) RubisCO forms and almost six times higher than *cbbL*-IA (avg 1.20 × 10^3^ genes, n = 103). The high levels of form II RubisCO, which are mostly found in the hypolimnion but with a significant amount also in the oxygen rich upper water column of the lake, emphasizes the fact that RubisCO form II is often present in bacteria that also have form I RubisCO^8^. Based on the dissimilar kinetic properties of different Rubisco forms, chemoautotrophs operating two or even three forms of RubisCO would enable these organisms to efficiently assimilate CO_2_ under varying aerobic conditions^34^. The presence of RubisCO form II in the oxygen-saturated pelagic zone can also be explained by the presence of particles and aggregates that provide favorable conditions established by oxygen-depleted microenvironments^35^. Based on a previous study including Piburger See, the affiliation of *cbbM* sequences to a wide range of different functional groups allows only a limited understanding on the specific metabolisms and associated ecophysiology of bacteria harbouring this form of RubisCO^6^.

### Lack of Thaumarchaeota using the HP/HB cycle

The seasonal distribution of genes coding for 4-hydroxybutyryl-CoA dehydratase (*hcd*), the key enzyme in the HP/HB cycle used by nitrifying Thaumarchaeota, was characterized by the absence or very low levels of *hcd* genes throughout the year (Fig. 2f). Maximum *hcd* abundances were observed in September at depths between 9 and 15 m (24–33 genes ml^−1^, Fig. 2f). In agreement with *hcd* as autotrophic gene marker, also *amoA* genes coding for thaumarchaeal ammonia monooxygenase were rare or close to the detection limit (Fig. 2g). Highest numbers of *amoA* genes were also detected between 6 and 15 m in September (13–27 genes ml^−1^). The absence of Thaumarchaeota in Piburger See was also reported based on sequence analysis of functional genes in the HP/HB cycle^6^ and other smaller lakes based on qPCR analysis^7^, where samples were mostly taken during summer stagnation. The reason for the lack of Thaumarchaeota in upper water layers can be explained by surface-related environmental factors, including light inhibition, competition with phototrophs for inorganic carbon and nutrients and potential grazing pressure by mixotrophic organisms^6,7,23,25,36,37^. In contrast to deep lakes, where Thaumarchaeota are abundant nitrifiers in the oxygenated aphotic zone throughout the year^20,21,38,39^, the hypolimnion of Piburger See is characterized by low dissolved oxygen concentrations that overlap with inhibiting factors in the euphotic zone during summer. This situation could explain the absence of Thaumarchaeota over the entire water column during summer stagnation^7^. On the other hand, it has been shown that seasonality has a strong impact on Thaumarchaeota in the epilimnion of lakes, with generally higher abundances of Thaumarchaeota in the winter season than in the summer^37^. The absence of Thaumarchaeota in the entire water column of Piburger See during the ice-cover period does not confirm the original hypothesis of our study that during this period the pelagic zone of the lake represents a suitable habitat for Thaumarchaeota. The environmental reasons for their absence are unknown, however, methodological issues based on primer coverage and specificity are unlikely. Both primers, GenAOA and qPCR_hcd, were already evaluated in former studies^7,40^ (Table 1) and produced also highly similar results when applied for the quantification of *amoA* and *hcd* genes in Thaumarchaeota in eight different lakes^7^. Clearly, more research is required to elucidate which additional factors are responsible that Thaumarchaeota are also highly constrained under ice and why niches with potentially favourable conditions for nitrifying Thaumarchaeota are not realised during the winter months.

## Conclusions

Bacteria using the CBB cycle with different RubisCO forms showed distinct seasonal and depth-dependent distribution patterns. Cyanobacteria and obligate chemolithoautotrophs using form IA RubisCO are generally highly specialized in their physiology and these organisms developed strong adaptation to narrow temporal ecological niches related to seasonal changes in the lake. In contrast, variations in abundance and seasonality of RubisCO form IC genes, which are often found in facultative autotrophs, were less pronounced. This can be explained by a higher flexibility to changes in environmental conditions by bacteria using form IC RubisCO, based on their ability to utilize different carbon sources. Throughout the entire year, highest numbers of RubisCO Form II genes were measured under anoxic and microaerobic conditions in the hypolimnion of the lake, supporting the biochemical features of form II with a low specificity factor for CO_2_. Functional genes related to nitrifying Thaumarchaeota were characterized by levels close to the detection limit, which is in accordance with other studies, suggesting that Archaea using the HP/HB cycle are of minor importance in smaller lakes. The reason for the absence of Thaumarchaeota during winter months under ice, when environmental conditions are more favorable, remains unresolved. Overall, this study provides insight on how seasonal limnological changes in a dimictic lake are related to the distribution of prokaryotes based on an autotrophic perspective.

## Materials and Methods

### Study site and sampling

Piburger See is an oligo-mesotrophic soft water lake situated in a crystalline area in the Tyrolean Alps, Austria (47°11′42″N, 10°53′20″O, 913 m a.s.l.). The lake is dimictic and usually ice-covered from mid-December until the end of March. The catchment is composed of coniferous forest (60%), grazing land and bare rocks (35%) as well as agriculture (5%). The lake has an area of 0.17 km^2^ and the water retention time is about two years. Detailed information on the lake can be found elsewhere^31,41^. Water samples were taken monthly over a period of one year (from Oct. 2016 to Sep. 2017) along a vertical profile at 3-m intervals (nine samples from 0 to 24 m depths) at the deepest part of the lake (24.6 m). Samples were collected with a modified Schindler-Patalas sampler (UWITEC, Mondsee, Austria). Depth profiles of temperature, pH, conductivity, and oxygen were determined with a multi-parameter probe (YSI model 6600; Yellow Springs Instruments, USA). In addition to probe measurements, oxygen concentrations were quantified at sampling depths using the Winkler titration method. Secchi transparency was also determined. Lake water nutrients were measured following standard techniques: major cation and anion compositions of water samples were quantified by ion chromatography (Dionex DX-120, Dionex Inc., Sunnyvale, USA). Dissolved organic carbon (DOC) and total organic carbon (TOC) were measured using a TOC/TN-Analyzer (TOC-5000, Shimadzu, Kyoto, Japan). Chlorophyll *a* was measured spectrophotometrically according to Jeffrey and Humphrey^42^.

### DNA-extraction and quantitative PCR

For DNA analyses, 480–1065 ml of lake water were filtered on polyethersulfone filters (pore size 0.22 µm, Millipore, Bedford, USA) and stored at −20 °C until use. DNA extraction was performed with a PowerWater©DNA Isolation Kit (Qiagen Inc., Hilden, Germany) according to the protocol of the manufacturer. The DNA amount was measured fluorometrically (Quantus^Tm^, QuantiFluor®dsDNA chemistry, Promega Corporation, Fitchburg, USA). Quantitative PCR was accomplished using a ddPCR system (QX200™, Bio-Rad Laboratories, Hercules, USA). Droplet generation was performed with a robotic droplet generator (AutoDG™ Instrument, Bio-Rad). Reactions for ddPCR are based on a QX200 ddPCR EvaGreen Supermix (Bio-Rad), set up to a final volume of 22 µl in 96-well plates following the instructions of the manufacturer. Primers used for ddPCR include genes coding for the enzyme 4-hydroxybutyryl-CoA dehydratase in the thaumarchaeal HP/HB cycle and different forms of RubisCO in the CBB-cycle were designed in former studies^6,7,30,34,43^. Primers targeting RubisCO form IC genes related to *Burkholderiales* in lakes were designed for this study (Table 1). Optimal annealing temperatures and primer concentrations for ddPCR were determined based on temperature gradient experiments and testing different primer concentrations. Droplet generation was performed following the protocol provided by the manufacturer (Bio-Rad). For PCR, plates were sealed by heat and PCR amplification was performed using a standard thermal cycler (T100, Bio-Rad). The PCR reaction included the following cycling conditions: initial step of 5 min at 95 °C, 37 cycles including 30 s at 95 °C, 30 s of primer annealing at primer specific temperatures (see Table 1) and 2 min at 72 °C. The final step includes incubation temperatures of 4 °C for 5 min and 90 °C for 5 min for signal stabilization of the PCR. A ramp rate at 2.5 °C/sec was used. Droplet signal measurements were performed using a droplet reader (QX200, Bio-Rad) following the protocol of the manufacturer. Data were analyzed with the software QuantaSoft™ 1.7.4. (Bio-Rad). Samples containing over 100,000 copies were diluted 1:10 or 1:100 and analyzed again. The reliability of the automated threshold settings and fluorescence amplitudes of positive and negative droplets were examined visually.

### Evaluation and sequencing of newly designed RubisCO primers q_cbbL_IC-lake

The specificity and coverage of the newly designed qPCR primer pairs q_cbbL_IC-lake (Table 1) were tested using DNA extracts obtained from different lakes for sequence analysis (Fig. 3). Standard PCR-reactions were separated on 1.5% agarose gels^6^. DNA of proper size was cut out of the gel and purified (MinElute® Gel Extraction Kit, Qiagen Inc.). PCR products selected for cloning were ligated into pGEM-T-Easy Vector plasmid (Promega Corporation) and transformed into JM109 competent cells^6^. Plasmids were screened for the presence of inserts with the proper length by PCR (GoTaq® G2 Hot Start Master Mix, Promega) using vector-specific primers M13 and inspection of DNA bands on an agarose gel^6^. Selected PCR-products were Sanger sequenced by a sequencing service enterprise (Eurofins MWG Operon, Ebersberg, Germany). Closest relatives to nucleotide sequences and deduced amino acid sequences were obtained using NCBI’s sequence similarity search tools BLASTN and BLASTP (https://blast.ncbi.nlm.nih.gov/Blast.cgi). Deduced amino acids were aligned using MUSCLE algorithm as implemented in MEGA X version 10.0.04 software package^44^, followed by a visual inspection of the alignment. Neighbor-Joining trees applying gamma distribution as the distance method were also computed with the MEGA X program. Bootstrap analysis (1000 replicates) was used to obtain confidence estimates for tree topology. The phylogenetic tree was condensed by compressing subtrees with highly similar sequences.

### Statistical analysis

Redundancy analysis (RDA) was used to test and visualize the influence of different environmental factors on the spatiotemporal distribution of functional genes for CO_2_ fixation in the lake. A forward selection procedure, based on the Monte Carlo permutation test, was used to select meaningful explanatory variables from all environmental parameters measured. The choice of data includes only environmental parameters with a p-value < 0.02.

### Sequence data deposition

RubisCO form IC sequences data obtained with primers q_cbbL_IC_lake have been submitted to GenBank databases under accession numbers MK907609 - MK907675.
